# Beating Cancer-Related Fatigue With the Untire Mobile App: Protocol for a Waiting List Randomized Controlled Trial

**DOI:** 10.2196/15969

**Published:** 2020-02-14

**Authors:** Simon S Spahrkäs, Anne Looijmans, Robbert Sanderman, Mariët Hagedoorn

**Affiliations:** 1 Department of Health Psychology University Medical Center Groningen University of Groningen Groningen Netherlands; 2 Department of Psychology, Health & Technology University of Twente Enschede Netherlands

**Keywords:** RCT, mHealth, app, intervention, fatigue, quality of life, cancer patients, cancer survivors, psycho-oncology

## Abstract

**Background:**

Many cancer patients and survivors worldwide experience disabling fatigue as the main side effect of their illness and the treatments involved. Face-to-face therapy is effective in treating cancer-related fatigue (CRF), but it is also resource-intensive. Offering a self-management program via a mobile phone app (ie, the Untire app), based on elements of effective face-to-face treatments, might increase the number of patients receiving adequate support for fatigue and decrease care costs.

**Objective:**

The aim of this protocol is to describe a randomized controlled trial (RCT) to assess the effectiveness of the Untire app in reducing fatigue in cancer patients and survivors after 12 weeks of app use as compared with a waiting list control group. Substudies nested within this trial include questions concerning the reach and costs of online recruitment and uptake and usage of the Untire app.

**Methods:**

The Untire app study is a waiting list RCT targeting cancer patients and survivors who experience moderate to severe fatigue via social media (Facebook and Instagram) across 4 English-speaking countries (Australia, Canada, the United Kingdom, and the United States). The Untire app includes psychoeducation and exercises concerning energy conservation, activity management, optimizing restful sleep, mindfulness-based stress reduction, psychosocial support, cognitive behavioral therapy, and physical activity. After randomization, participants in the intervention group could access the Untire app immediately, whereas control participants had no access to the Untire app until the primary follow-up assessment at 12 weeks. Participants completed questionnaires at baseline before randomization and after 4, 8, 12, and 24 weeks. The study outcomes are fatigue (primary) and quality of life (QoL; secondary). Potential moderators and mediators of the hypothesized treatment effect on levels of fatigue and QoL were also assessed. Link clicks and app activation are used to assess reach and uptake, respectively. Log data are used to explore the characteristics of app use. Sample size calculations for the primary outcome showed that we needed to include 164 participants with complete 12-week measures both in the intervention and the control groups. The intention-to-treat approach is used in the primary analyses, which refers to analyzing all participants regardless of their app use.

**Results:**

Participants were recruited from March to October 2018. The last participant completed the 24-week assessment in March 2019.

**Conclusions:**

This mobile health (mHealth) RCT recruited participants online in multiple countries to examine the uptake and effectiveness of the Untire self-management app to reduce CRF. Many advantages of mHealth apps are assumed, such as the immediate access to the app, the low thresholds to seek support, and the absence of contact with care professionals that will reduce costs. If found effective, this app can easily be offered worldwide to patients experiencing CRF.

**Trial Registration:**

Netherlands Trial Register NL6642; https://www.trialregister.nl/trial/6642.

**International Registered Report Identifier (IRRID):**

DERR1-10.2196/15969

## Introduction

### Background

The most common and distressing long-term side effect of cancer and its treatment is cancer-related fatigue (CRF) [1-3]. CRF can be defined as “a distressing and persistent subjective sense of physical, emotional, and cognitive tiredness or exhaustion related to cancer or cancer treatment that is not proportional to recent activity and interferes with daily functioning and is not relieved by sleep or rest” [3,4]. A third of the patients with cancer suffer from fatigue on a daily basis [5,6], which can persist for up to 10 years after diagnosis [4] with a considerable impact on their lives [2]. Besides the personal impact of CRF, the economic impact of CRF is substantial. A recent European health economics study has indicated that over 75% of patients with cancer who were employed changed their employment status, and 28% stopped working. A US study reported that 30% of patients reduced their work hours, with an overall average of 4 days per month per patient [5].

Most patients with cancer (74%) and oncologists (80%) perceive fatigue as a symptom impossible to manage or to treat successfully [7]. Oncology practice lacks an adequate approach to address the consequences of CRF. Usually, physicians inform patients with cancer about the risks of cancer treatment, including the possibility of becoming fatigued [8]. However, patients assume that treatment for CRF is not available or not effective, which often discourages patients from asking for help. Also, when physicians make recommendations to assist patients in addressing CRF, these recommendations tend to be nonspecific as 40% of physicians recommended doing nothing, followed closely by prescribing rest (37%). About 9% of the physicians in this situation used prescription drugs, which regularly have severe adverse effects [8]. The inadequate provision of care is not solely due to the lack of education about fatigue treatment, but also due to the complex origin and underlying mechanisms of fatigue. The etiology of CRF is multidimensional, involving physiological, biochemical, and psychological systems [9].

When it comes to the psychosocial treatment of CRF, therapist-guided face-to-face interventions have shown to be effective [10]. Such therapies include energy conservation, activity management, optimizing restful sleep, mindfulness-based stress reduction (MBSR) techniques, psychosocial support, and cognitive behavioral therapy (CBT) [4,11,12]. However, face-to-face treatment approaches are limited in reach and resource-intensive, as 1 therapist can only treat 1 patient or patient-group at a time. Therapist-guided electronic health (eHealth) interventions in which health care is delivered over the internet via technologies are offering a more flexible and low-threshold approach [13]. Another advantage of eHealth is that due to Web-delivery, also patients with little time and energy can take part in the treatment [14,15]. One example of a therapist-guided eHealth intervention to reduce fatigue in cancer survivors is the *More fit after Cancer* (*Fitter na Kanker* in Dutch) intervention in which patients received Web-based mindfulness-based cognitive therapy via a psychologist. This intervention showed strong evidence in reducing fatigue in survivors of cancer [13]. Although eHealth interventions involving therapist-guidance can be offered effectively over the internet reaching many patients worldwide, these interventions are still limited by the availability of specialized psychologists.

One way to reach many patients worldwide is to make use of self-management interventions delivered via the mobile phone (mobile health [mHealth], which is a subbranch of eHealth) in the form of an app. Advantages of mHealth apps concern the reach (ie, apps are widely and easily accessible by many patients), the low threshold intensity to engage in apps as compared with face-to-face treatment, and the low resource-intensity of the care provided (ie, it does not require input from a therapist as it can function as a self-management tool) [16]. As mHealth permits treatment of participants worldwide without having contact with health care professionals, online recruitment might be an effective way to reach out to a large number of potential mHealth users within a short time [17].

Inspired by the academic research and clinical practice from the Helen Dowling Institute (HDI) for psycho-oncology including the aforementioned *More fit after Cancer* trial, the start-up Tired of Cancer BV (Utrecht, The Netherlands) developed the so-called Untire app, with the goal to deliver an effective self-management tool to improve CRF and quality of life (QoL) of patients and survivors of cancer who feel fatigued. The Untire app is built for smartphones and tablets for both mobile operating systems iPhone (Apple Inc) and Android (Google). The intervention is based on the aforementioned successful elements of face-to-face therapy for CRF, that is, energy conservation, activity management, optimizing restful sleep, MBSR, psychosocial support, CBT, and physical activity exercises [9] and in line with the guidelines of the National Comprehensive Cancer Network [18]. The app aims to create awareness by providing psychoeducation, to give insight into one’s energy levels, thoughts and behaviors about fatigue, and to help users to challenge unhelpful thoughts (eg, catastrophizing thoughts) and behaviors (eg, increase physical activity) by means of exercises. It is hypothesized that creating awareness and adjusting unhelpful thoughts and behaviors will lead to improvements in CRF and QoL. The current international study aims to examine whether the use of the Untire app has the potential to reduce CRF and improve QoL in patients and survivors of cancer. Until 2017, as far as we know, no mHealth interventions were developed to decrease CRF in patients with cancer and survivors [4,19].

### Aims of the Research

The primary aim of this waiting list randomized controlled trial (RCT) is to assess the effectiveness of the Untire app in reducing fatigue in patients and survivors of cancer after 12 weeks of app access as compared with patients in a waiting list control group.

Secondary aims are to assess the effectiveness of the Untire app in improving QoL in patients and survivors of cancer after 12 weeks of app access as compared with patients in a waiting list control group; to examine the reach and costs of online recruitment, study drop-out, and uptake and usage of the Untire app; and to determine which factors moderate (ie, age, gender, and country) and mediate (ie, mindfulness, physical activity, depression, sleep, pain interference, and fatigue catastrophizing) the hypothesized effect of the intervention.

## Methods

### Approval

The Untire app study has been approved by the Medical Ethical Committee of the University Medical Center Groningen (UMCG), the Netherlands. Hereafter, the study received either ethical approval or a waiver from authorized institutions in the 4 English-speaking countries targeted (ie, Australia, Canada, the United Kingdom, and the United States; see Multimedia Appendix 1 for a detailed overview of ethical review and approval by country). The trial has been registered on 29/11/2019 on the Netherlands Trial Registry (NL6642).

### Study Design and Setting

**Figure 1:**
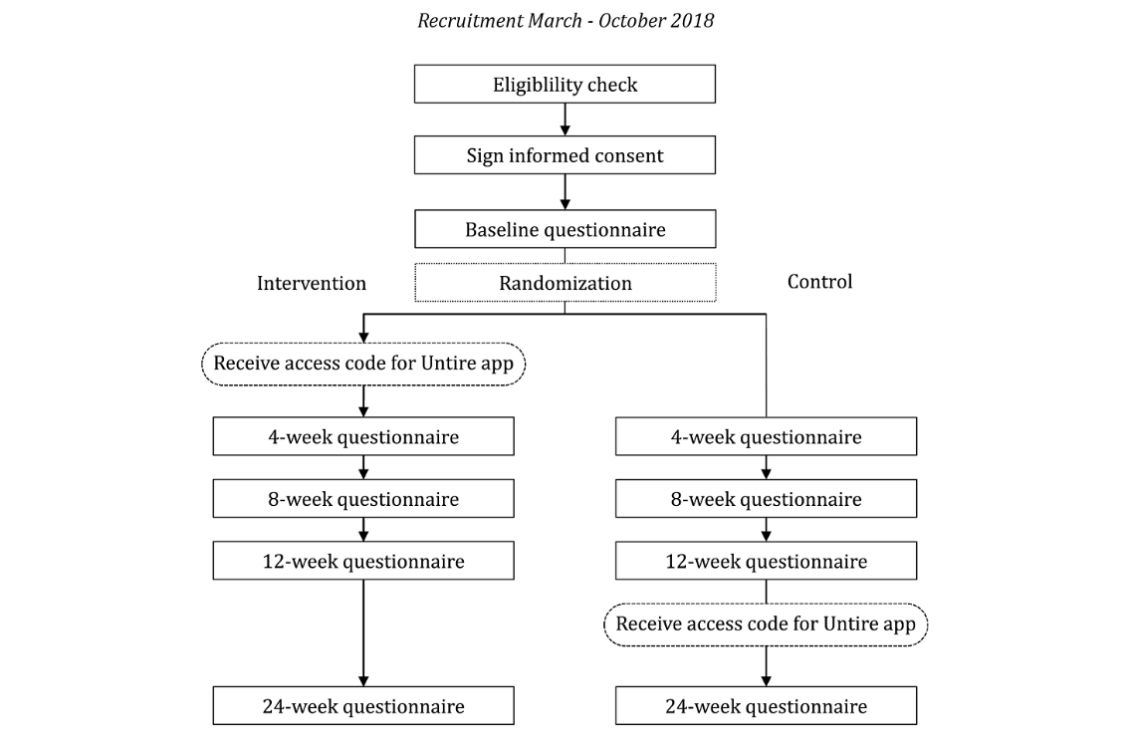
Study design to test the effectiveness of the Untire app in 4 English-speaking countries.

The Untire app study is a large-scale international waiting list RCT aimed to examine the effectiveness of the Untire app in improving CRF and QoL in patients and survivors of cancer. The Untire self-management app was launched worldwide from March 2018, where study enrollment took place until October 2018. Also, after the recruitment period, the app remained available. Participants randomly allocated to the intervention group received the Untire app at the start of the trial (Figure 1). After 12 weeks, also the control participants received the intervention (ie, 1 year of free app access). During the waiting time, control participants were allowed to seek or accept other treatments for their fatigue. The eligibility check and all other assessments, including demographics of participants, were carried out by using Questback’s Web-based questionnaire system *EFS-survey*.

### Study Population

The Untire app study targeted patients and survivors of cancer of 18 years or older, who experienced persistent fatigue at moderate or severe levels (ie, an average composite score of >3 on items 1, 2, and 3 of the Fatigue Symptom Inventory (FSI) [20,21]) and owned a smartphone, tablet, or iPad. Exclusion criteria were a diagnosis of and receiving treatment for a severe mental disorder (ie, major depression, psychotic disorder, anxiety disorder, or addiction) as these persons may need more intensive treatment than offered by the app. Also excluded were persons having a diagnosis of chronic fatigue syndrome, myalgic encephalomyelitis, or fibromyalgia as the app focusses on improving physical activity, which is not recommended and could potentially be harmful to these patients [22].

### Procedure and Recruitment

We approached potential participants over the internet via social media advertisement campaigns (Facebook and Instagram). Multiple advertisement campaigns were created to promote the link to the study page. Both female and male patients and survivors of cancer of all age groups >18 years, and who have shown interest in topics related to cancer, fatigue, and cancer survivorship were targeted in the first advertising campaigns. On the basis of participant characteristics of those who showed interest (ie, clicked on the advertisement link), comparable participants (ie, look-alike audiences) were approached in subsequent advertisement campaigns.

Interested persons were directed to the landing page of the Web-based survey tool (ie, Questback) that was used for data collection. Participants were screened for eligibility, and if eligible, they received an information letter and gave digitally informed consent. Hereafter, participants completed a baseline questionnaire, after which they were randomized into the intervention or control group. Next, participants received an email with the information letter (see Multimedia Appendix 2) and their digital signed consent (see Multimedia Appendix 3). Intervention participants received a second email with a unique access code that could be used to activate the Untire app after downloading it from the app store, enabling free usage. Subsequently, participants received email invitations and reminders at 4, 8, 12, and 24 weeks to fill in the questionnaires. Once control participants completed the 12-weeks survey, they also received an email with a unique access code that could be used to activate the app after downloading it from the app store, enabling free usage. The 24-weeks follow-up assessment was used to assess maintenance of the expected decline in fatigue in the experimental group. Assessments did not last longer than 15 min.

#### Randomization and Blinding

Participants who completed the baseline questionnaire were randomized 2:1 by the Web-based academic survey tool (ie, Questback) into the intervention and control groups. The randomization ratio was chosen for two reasons. First, we expected that intervention participants were less likely to complete the 12-weeks assessment after receiving the incentive for participation (ie, the free access to the Untire app) already at baseline, as compared with control participants who received the incentive after completing the 12-weeks measure. The 2:1 randomization increased the likelihood of a balanced number of completed 12-weeks measures. Second, in case the drop-out rate in the intervention group was higher than expected at the 12-weeks measures, it was considered convenient to have large enough numbers in the intervention group for in-depth app use analyses. Due to the character of the intervention, it was not possible to blind participants and assessors to intervention or control allocation.

### Intervention

Participants in the intervention group were instructed to work through the app at their own pace and could freely choose modules to work on. Although daily use of the Untire self-management app was recommended, participants were instructed to use the app at least once a week. Users who had not been active for 5 days were sent a reminder. Users who opened the app at least seven times within 10 days received a rating pop-up (ie, to assess satisfaction and ask for feedback).

**Figure 2:**
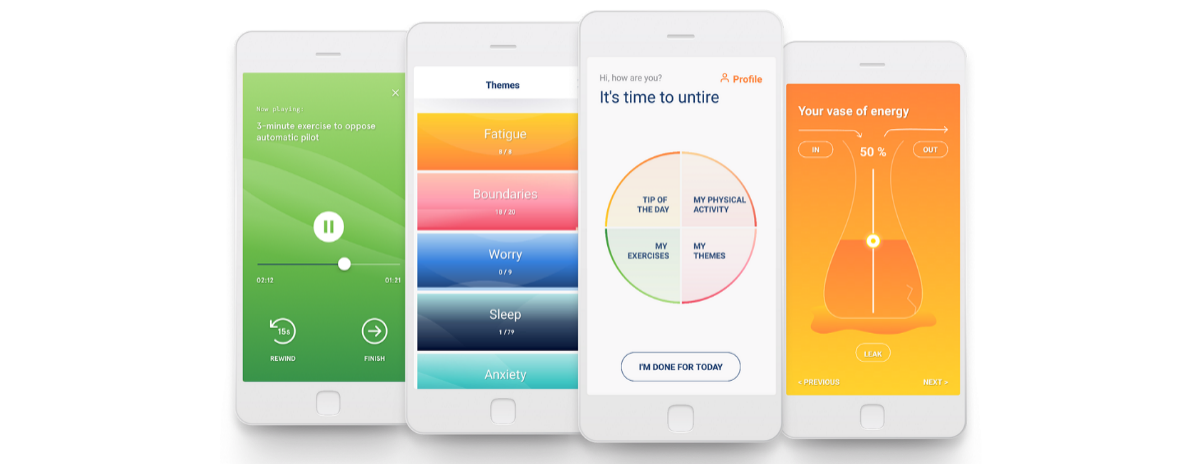
The Untire self-management app: (a) mindfulness-based stress reduction audio exercises, (b) psychoeducative themes, (c) the daily program, and (d) the vase of energy.

After opening the Untire app for the first time, participants received a short introduction on how to use the app. Participants were informed that every time they revisit the app (ie, reopen the app), a (new) daily program will be presented. The daily program consists of the 4 following core components: (1) *My themes*, (2) *My exercises*, (3) *Tip of the day*, and (4) *My physical activity* (Figure 2). Participants also received information about the weekly assessments of their fatigue and related components, the so-called *Quick scan*. The *Vase of Energy* is an integral part of the Quick scan. Participants were also invited to think about informing a friend or family member, a so-called *buddy*, to help or motivate them to work with the app. All the modules are explained below in detail (also see Table 1 and Figure 2).

**Table 1:** Daily and weekly Untire app components, description, and use.

Untire app components			Description
**Daily program**			
	**My themes**		Receive psychoeducation and exercises on topics such as fear, worry, anxiety, and sleep.
		Basics	Get basic education about different sources of fatigue (ie, the other themes) and receive an overview of how to work with the Untire app, including the rationale of the *vase of energy*.
		Fatigue	Receive psychoeducative and medical background information and exercises about cancer-related fatigue.
		Anxiety	Receive information on the physiology and the harmlessness of anxiety, as well as several tips on how to cope with fear.
		Worry	Learn to distinguish between thoughts and facts, and learn that thoughts are malleable. Furthermore, participants receive challenging questions to explore their thoughts.
		Boundaries	Learn to accept that energy levels are often lower as compared with before your disease. Furthermore, learn to understand that time-outs may be needed and that you can actively manage your boundaries. Gain insight into your boundaries to prevent crossing them repeatedly.
		Sleep	Learn about different factors related to sleep problems and the vicious circle of insomnia, which may influence (quality of) sleep. Receive suggestions on how to improve your sleeping behavior.
		Self-care	Improve your self-compassion by receiving 20 suggestions for self-care.
		Nutrition	Receive general information on healthy nutrition based on the latest scientific research.
	My exercises		Engage in 17 different mindfulness-based stress reduction exercises to identify, manage, and release stress (ie, *attention to the breath* or body scan).
	**Tip of the day**		Tips are ideas or quotes based on positive psychology, which should help to get into a good mood (eg, *Gratitude*).
		Eg, gratitude	For instance, receive a gratitude writing tip and exercise, in which you write down 3 moments of the day that you felt grateful. The gratitude tip will support you in adopting a positive thinking style.
	**My physical activity**		Receive information and exercises on 3 different aspects of the Untire physical exercise training program to increase levels of physical activity and muscle strength.
		Planning	Receive several exercises and tips to deal with limited levels of energy, such as agenda taking and indicate how the upcoming days will be about activities and energy levels.
		Build up energy	Build up your energy by carrying out daily physical activity based on guidance in 30 min a day (daily increasing difficulty). Receive guidance in improving sedentary behaviors through information, exercises, and tips.
		Power	Receive guidance to build up power and muscle strength through information, exercises, and tips.
**Assessments**			
	**Quick scan**		Receive weekly evaluations involving 4 questions about well-being as well as 3 questions about energy (part of the *vase of energy*). Via the personal profile, you can see your progress anytime.
		Fatigue	How was your fatigue last week?
		Burden	How was your burden last week?
		Happiness	How happy have you been last week?
		Satisfaction	How satisfied have you been last week?
		Vase of energy	Receive 3 questions presented visually by the vase of energy: What gives you energy? What costs you energy? What is your energy leak? The weekly pattern provides insights into energy levels.

#### My Themes

My Themes is 1 of the 4 core components of the Untire app, which serves the overarching purpose of providing patients and survivors of cancer with background information about various aspects of their CRF. Psychoeducation is known as a crucial element of face-to-face therapy for patients with CRF [4]. Themes that are often associated with CRF include fatigue, anxiety, worry, boundaries, sleep, self-care, and nutrition. Participants started with a theme called Basics, which taught them essential introductory information on CRF and explained the rationale behind the Vase of Energy. Hereafter, participants could choose the themes based on their interests. The themes mostly consist of articles to read or listen to (audio) and small related exercises (eg, listing realistic activities).

#### My Exercises—Mindfulness-Based Stress Reduction

Stress-reduction exercises involving relaxation techniques and meditation exercises are often used in mindfulness-based cognitive therapy for patients and survivors of cancer (Table 1) as reduced stress is generally associated with a reduction of CRF [4,23-26]. Participants are offered 1 of 17 stress-reduction exercises. Participants could choose to do a new exercise or also redo earlier completed exercises. *My exercises* include stress-reducing mind-body activities such as breathing techniques and body scans. All exercises are audio-guided.

#### Tip of the Day

Tips are provided to foster insights and tricks to improve the daily mood of patients and survivors. One example is the tip of the day called *gratitude*, in which participants are instructed to write down 3 aspects that they are feeling genuinely happy about that day. Participants are instructed to continue this exercise for the upcoming days. Every day the tip component is revisited, a new tip is displayed and accompanied by a motivational image or catchphrase.

#### Physical Activity

The physical activity part aims to increase participants’ physical activity levels and muscle strength and, even more important, gives insight into the management of energy levels and activities. The app teaches 3 methods to improve the participant’s energy balance, supported by video-guided strength exercises (Table 1). Positive effects of physical activity and gaining fitness are explained, and participants learn that, even though they feel tired, exercise might improve their feeling of tiredness. Also, a schedule is presented as an example of how to build up to 30 min of physical activity a day, which is the recommended duration of physical activity per day [27].

#### Quick Scans

Apart from the 4 core components, every week, participants are assessed on their level of fatigue and related aspects in a weekly quick scan. The quick scan aims to evaluate to what extent participants experienced fatigue and related aspects in the previous week. In this way, participants are expected to gain insights into their patterns and progress of fatigue and energy levels over time. The quick scan is solely for informing participants; the app does not offer any personalized feedback based on participants’ answers. The quick scan consists of 4 items assessing the level of fatigue, fatigue burden, satisfaction, and happiness on a visual analog scale: *How was your fatigue this last week?* *How was your burden of fatigue this last week*? *How satisfied with life have you been this last week? How happy have you been this last week?* Every week, participants have to fill in once their *Vase of energy.* The vase of energy is a visual presentation tool helping participants to track and visualize personal energy levels. Participants gain insights into factors leading to a reduction or increase in energy levels. Apart from that, so-called energy leaks are explored, which can pertain to a constant drain (eg, pain, anxiety, and stress). Participants indicate what was lowering their energy levels, as well as what was giving them energy and what caused a consistent energy leak.

#### Content Delivery Forms

The content of the Untire app is presented in different forms. Patients and survivors of cancer can read educative articles, but often also an audio option is available, as listening to the material instead of reading it might be less tiring for patients with CRF. Besides reading texts and listening to audio texts or exercises, the Untire app contains numerous video animations aimed to explain complex aspects and exercises in an easy to understand and visually attractive way. Apart from the form of content delivery (ie, text, audio, and video), also persuasive elements (eg, motivational pictures in between blocks of text) are used to visualize key aspects (eg, the biological clock for sleeping quality). Other persuasive elements include progression bars, rewards, badges, and constant encouragements. To encourage participants to use the app at their own pace, they receive gentle reminders to take a break after going through 5 pages within a theme. The app fosters social commitment by supporting participants to invite a so-called *buddy*. In this way, participants can manage their fatigue together with a family member or friend. Furthermore, participants are also invited to join the Untire community on Facebook, which provides a platform for the exchange of thoughts and allows socializing in a broader social environment.

#### App Version

The study used version 2.1 of the app, which has been developed based on a pilot and feasibility study with version 1.0, and a usability study with 19 users and 9 professionals focusing on the content, use, and technical aspects of version 2.0. For version 2.1, improvements were made in the onboarding system, tone-of-voice, and presentation of content. For the improvements in the onboarding system, an additional 5 users participated in a UX usability test. During the study, only minor technical updates and changes (ie, mostly bug fixes) have been made.

### Outcome Assessment

Data in this study have been collected by means of Web-based questionnaires and in-app log data. Table 2 presents a complete overview of the variables assessed by the Web-based questionnaires at each measurement wave for the intervention and control participants, respectively. Variables were assessed by either using validated questionnaires, using a selection of items of a validated questionnaire, or by items specifically created for this trial. We realize it may be a burden for patients with CRF to fill out all these assessments. To enhance patients’ commitment to the study and to reduce attrition, we carefully selected a limited number of questionnaires and items to include.

**Table 2:** Questionnaires to assess the primary outcome, secondary outcomes, moderators, mediators, and explanatory factors (including background attitudes, experiences of app use, and cost-effectiveness).

Variables				Measurement (number of items)	Measurement in weeks					
					0 (baseline)	4	8	12		24
**Primary outcome measure**										
	Fatigue symptoms			FSI^a^ (15): Severity Scale (4) and Interference Scale (7)	I^b^C^c^	IC	IC	IC	IC	
**Secondary outcome measures**										
	QoL^d^			EORTC-QLQ-30^e^—Past week (1) and QoL—On Average (1)	IC	IC	IC	IC		IC
**Moderators**										
	Demographic and health characteristics			—^f^	IC	—	—	—		—
**Mediators**										
	Mindfulness			MAAS-5^g^ (5)	IC	IC	IC	IC		IC
	Physical activity			(3)	IC	IC	IC	IC		IC
	Depression			PROMIS-29^h^ (4)	IC	IC	IC	IC		IC
	**Sleep**									
		Quality		PROMIS-29 (1)	IC	IC	IC	IC		IC
		Disturbance		SLC-90-R^i^ (3)	IC	IC	IC	IC		IC
	Fatigue catastrophizing			FCS^j^ (13): Rumination (4), Magnification (3), and Helplessness (6)	IC	IC	IC	IC		IC
**Explanatory factors**										
	Patients expectancy			(3)	I	—	—	C		—
	Patients motivation			(2)	I	—	—	C		—
	Patients support in environment			WHYMPI/MPI^k^ (5)	IC	IC	IC	IC		IC
	Pain interference			RAND-36^l^ (2)	IC	IC	IC	IC		IC
	Experienced changes			(5)	—	—		IC		IC
	User experience of the app			(19)	—	—	—	I		C
	Cost-effectiveness			(6)	—	—	—	IC		—

^a^FSI: Fatigue Symptom Inventory (fatigue severity scale, fatigue interference scale).

^b^I: intervention measurement.

^c^C: control measurement.

^d^QoL: quality of Life.

^e^EORTC-QLQ-30: European Organization for Research and Treatment for Cancer Quality of Life Questionnaire.

^f^Not applicable.

^g^MAAS-5: The Mindful Attention Awareness Scale.

^h^PROMIS-29: Patient-Reported Outcomes Measurement Information System (depression scale, sleep scale).

^i^SLC-90-R: Symptom Checklist-90-Revised (sleep disturbance scale).

^j^FCS: Fatigue Catastrophizing Scale (fatigue catastrophizing—adapted from PCS: Pain Catastrophizing Scale; rumination, magnification, and helplessness scales).

^k^WHYMPI/MPI: The West Haven-Yale Multidimensional Pain Inventory.

^l^RAND-36: Research and Development (pain interference scale).

#### Primary Outcome Measure

The primary outcome is the change of fatigue severity and fatigue interference from baseline to 12 weeks, which was assessed with the self-report questionnaire FSI [20,21]. Fatigue severity was assessed by calculating the average of 3 severity items (FSI items 1-3: *Rate your average level of fatigue during the past week; Rate your level of fatigue on the day you felt most fatigued during the past week; Rate your level of fatigue on the day you felt least fatigued during the past week*) on an 11-point Likert-scale ranging from 0 (not at all fatigued/no interference) to 10 (as fatigued as I could be/extreme interference). Fatigue interference was assessed by calculating a composite score of the average of 7 interference items (FSI items 5-11: *Rate how much, in the past week, fatigue interfered with your general level of activity, ability to bathe and dress, normal work activity [includes both work outside the home and housework, ability to concentrate, relations with other people, enjoyment of life, mood]*). A higher score indicates stronger severity or interference.

#### Secondary Outcome Measures

The secondary outcome is change in QoL from baseline to 12 weeks, which was assessed with the 1-item European Organization for Research and Treatment of Cancer Quality of Life Questionnaire (*How would you rate your overall quality of life during the past week?* [28]) as well as a self-constructed item of overall QoL (Overall QoL: *How would you rate your overall quality of life on average?*) on a 7-point Likert-scale ranging from 0 (very poor) to 7 (excellent). A higher score indicates a better QoL.

#### Moderating Factors

Demographic data were gathered on age, gender, country of residence, level of education, employment status, relationship status, and illness-related characteristics, such as being a patient or survivor of cancer, time since diagnosis, and type of treatment received.

#### Mediating Factors

The following potential mediating factors were assessed: mindfulness, physical activity, depression, sleep, and fatigue catastrophizing. Mindfulness was measured with the Mindful Attention Awareness Scale [29]. To measure physical activity, we constructed 3 questions regarding engagement in light, moderate, and vigorous physical activities. Depression was measured with the depression subscale of the Patient-Reported Outcomes Measurement Information System (PROMIS-29) [30]. Sleep was measured with the 1-item sleep quality subscale of the PROMIS-29, and 3 items of the sleep disruption subscale of the Symptom Checklist-90-Revised [31]. The fatigue catastrophizing scale was adapted from the Pain Catastrophizing Scale [32], and measured by the rumination, magnification, and helplessness subscales.

#### Explanatory Factors

Explanatory factors were included as these were assumed to be potentially helpful for the interpretation of the study results. Patients’ attitudes were assessed with questions about their expectations, motivations, and experienced support in their environment. Furthermore, as a proxy of impaired physical functioning, pain interference was assessed. Pain interference was measured with 2 items of the Research and Development Health Survey [33]. Perceived changes in levels of fatigue, overall QoL, physical activity, mood, and sleep were assessed with 1-item questions. User experience with the app was assessed to explore patients’ attitudes toward and experiences with the Untire app. Cost-effectiveness was assessed to explore the costs and benefits of the Untire app (see Table 1).

#### In-App Log Data

Log data are used to explore the characteristics of app use. Once participants activated their unique access code and gave consent to the privacy terms in the Untire app, participants were registered, and their log data were stored anonymously. Log data are automatically stored data about the activities and assessments (ie, Quick scans and the Vase of Energy) that participants perform, and registers the date on which activities and assessments were performed. App use can be described based on (1) the duration of app use in days and (2) the degree of app use. Log data will indicate the first, and the last time a participant completed an activity and the number of days between the first and last activity will be used as a proxy for the duration of app use. The number of completed activities will be counted and will be used as a proxy for the degree of app use, with a higher number of completed activities indicating more app use.

#### Data Management

Data collection took place over the internet on the secured and password-encrypted server of Questback. Anonymous back-ups were saved on the secured and encrypted server of the UMCG, permitting only access to the involved researchers.

### Statistical Analyses

#### Preparatory Analyses

We will present baseline characteristics (ie, demographics, disease, and treatment characteristics) for each variable side by side for the intervention and control groups by the mean (standard deviation), or frequencies and percentages.

### Main Analyses

#### Does the Untire App Reduce Fatigue Significantly After 12 Weeks of App Access?

To examine a treatment effect of the app on fatigue, the data collected via Questback’s Web-based survey of fatigue severity and interference will be analyzed using General Linear Mixed Models (GLMM) for 4 repeated measures (baseline, 4 weeks, 8 weeks, and 12 weeks), and 2 groups (intervention and control), following the intention-to-treat (ITT) approach. The ITT approach refers to analyzing all participants, regardless of app use, and is the primary approach in all outcome analyses. Assumptions of linearity, homogeneity of variance as well as the normal distribution of residuals of the model will be tested. The individual measures of fatigue ratings over time (level 1) are nested within each patient (level 2). The default GLMM model without a country factor will be compared with a model with a country factor concerning the best model fit using the Bayesian information criterion.

On the basis of previous studies testing health-related apps targeting behavior [34-39], we estimated the percentage of participants that would lose interest in the app or study assessments, and, therefore, would be lost to follow-up (eg, the 12-weeks measurement), at 60%. For the data analysis, we will, therefore, employ a model that can take missing data into account (ie, GLMM). A comparison of the outcomes of the model run in the ITT approach and the outcomes of a model run among those who have completed at least the 12-week assessment (T12-completers) will demonstrate the robustness of the findings.

In addition to the model validation, we will carry out sensitivity analyses to determine whether the duration and the degree of app use are related to the degree of change in fatigue severity and interference over time. Outcomes of active users will be compared with less-active users. Active and less-active users are categorized according to the duration and degree of app use. The dose-response relationship of app use and treatment effects will be explored in a three-way interaction (time×group×activity level), using GLMM.

### Secondary Analyses

#### Does the Untire App Improve Quality of Life Significantly After 12 Weeks of App Access?

To examine the treatment effect of the app on QoL, the data derived from the 2 QoL questions will be analyzed following the same analytic procedure as described above in the main analyses.

#### What Are the Reach, Costs of Online Recruitment, and What Is the Uptake and Usage of the Untire App (Study)?

To explore the number of participants reached using Web-based social media campaigns, we will use the number of Web-based advertisement link clicks, leading to the study landing page. To determine the costs of recruitment, the number of link clicks will be compared with the budget spent on social media campaigns. We will calculate the total costs of recruitment, the costs per person who clicked the link, and the costs per person that completed the baseline (study) assessment. To examine how many participants completed the assessments, activated the app, and used the app in the first 12 weeks of the study, percentages and count data will be used. Log data will indicate how many participants activated their unique access code, the number of days a participant has been active in the app, and the number of activities performed.

#### Which Factors Moderate, and Which Factors Mediate the Hypothesized Effect of the Intervention?

Moderating factors (ie, age, gender, and country) of the intervention effect of the app on CRF and QoL will be explored as a three-way interaction (time×condition×moderator variable) using GLMM as described above to gain insights into whether the intervention effect varies among different groups of participants, for example, patient with cancer versus survivor. Moreover, we will examine via longitudinal mediation analysis, whether levels of mindfulness, physical activity, depression, sleep, pain interference, and fatigue catastrophizing mediate the hypothesized effect of the intervention.

### Sample Size

Sample size calculations for the primary outcome of fatigue showed that we needed to include 164 participants with complete 12-weeks measures in the intervention and the control group (total N=328) to detect a between-group difference in change from baseline to 12 weeks with a minimal effect size of η^2^=0.10 (alpha=.05, 1−beta=.95). The sample size was calculated using the simplest between/within-group comparison (F tests—repeated measures analysis of variance [with within-between interaction]) in G*Power 3.1. With the inclusion of 820 participants (410 intervention participants; 410 control participants) and an estimated drop-out of (492/820) 60.0%, 164 participants are expected to remain in the intervention and 164 in the control group at the 12-week primary outcome measurement. The total baseline completer sample size of N=820 can be taken as a conservative upper bound as the final analyzes will be carried out using GLMM. GLMM takes more information into account and, thus, requires a smaller sample size given the same power.

## Results

Throughout March and October 2018, patients with cancer and survivors (N=1137) were eligible and gave consent to participate in this trial. Of these, 847 participants started with the baseline questionnaire and were randomized into two conditions, following a 2:1 randomization with 545 participants assigned to the intervention group and 302 participants assigned to the control group. The last participants completed their 24-week assessment in March 2019.

## Discussion

### Principal Findings

This protocol describes an RCT to assess the effectiveness of the Untire app in reducing fatigue in patients and survivors of cancer after 12 weeks of app use as compared with a waiting list control group. Substudies nested within this trial include questions concerning the reach and costs of online recruitment and uptake and usage of the Untire app. There are advantages of stand-alone mHealth apps assumed, such as the immediate access to the app, the low thresholds to seek support via an app, and lower costs in supporting patients as compared with therapist-guided treatments.

The Untire app is based on a face-to-face therapy protocol, which was developed by the HDI in the Netherlands [25]. Although successful therapeutic elements have been used continuously over the years (eg, psychoeducation, CBT, physical activity, and mindfulness) [40], new ways of treatment delivery are increasingly being explored. For example, face-to-face treatment protocols have been effectively adapted to fit therapist-guided internet therapy [13]. More recently, the ingredients of the *More fit after Cancer* protocol were used to develop an adapted version in the form of a stand-alone app. The key therapeutic ingredients remained, but no supervision of a therapist is available. Apart from this shift to self-management, the app offers flexibility as the patients are allowed to choose from the entire library of education and exercises instead of following a structured week-by-week program. Although the app includes proven successful therapeutic elements, it is important to test its effectiveness as the delivery mode differs from previously tested interventions. If we do find the app to be effective, it can easily be offered worldwide to patients with cancer and survivors experiencing fatigue.

The findings will not only show whether or not the app is effective in reducing fatigue and improving QoL but will also provide more insight into the underlying mechanisms. Several routes may lead to a reduction in fatigue. For example, participants may gain vitality through improvements in the quality of their sleep, increases in physical exercise, and better management of their physical activities (eg, balancing activities throughout the day) [13]. Participants may also improve because they learn to better manage their unhelpful or catastrophizing thoughts about their fatigue, possibly through mindfulness [13]. Previous diary research has shown that such catastrophizing thoughts are associated with increases in negative affect and fatigue during the day [41]. This study will demonstrate whether improvements in these factors mediate hypothesized improvements in fatigue. Patients with specific problems concerning sleep or physical activity, or with respect to unhelpful and catastrophizing thoughts may benefit specifically from improvements in the respective areas.

### Strengths and Limitations

The design of the study has both strengths and limitations. Strengths are its international focus and large scale, which will not only provide insight into the effectiveness of the app but also valuable information on the costs of online recruitment across different countries, the reach of patients who are interested in mHealth as a form of treatment for fatigue, and the uptake of the app. Costs of online recruitment might be hard to predict, as Facebook advertising campaigns cannot be targeted to patients with cancer directly. Patients with cancer can only be targeted indirectly based on shared interests in the field of cancer (eg, diagnosis, impact, and treatment of cancer). Furthermore, on the basis of the number of patients to be included, we hope to be able to conduct moderator analyses to determine whether the hypothesized intervention effect varies among different groups of participants (eg, patients with cancer versus survivors of cancer) and the context in terms of country where the intervention is delivered. The study contains a self-report eligibility check to include only patients with moderate and severe levels of fatigue, as these patients are hypothesized to benefit from the intervention. A drawback of our online recruitment procedure is the lack of access to hospital records, meaning that we cannot validate medical information (eg, type, duration, and treatment of cancer). Another study limitation is that remotely conducted automated mHealth studies regularly show large attrition rates [42]. Although high drop-out rates and sparing app use may be a natural characteristic, as the choice to discontinue usage is uncomplicated easy owing to the impersonal nature of the Web-based study and app assessments. We hope to counteract this mechanism by sending personal reminder emails to reinvite participants to complete the respective study assessments. Finally, the waiting list RCT will help to demonstrate causality (ie, can patients benefit from treatment versus no-treatment) and control for potential spontaneous improvements during the study period, but we cannot disentangle the effects of therapeutic elements and common factors (eg, attention). A drawback of using waiting list control designs might be that it artificially inflates intervention effect estimates as control participants could be influenced by design to literally *wait-to-change* and, thus, do not improve [43]. Using an active control condition could overcome this shortcoming but was not possible as an equivalent Web-based intervention was not available, and a comparison with therapist-guided treatment was not feasible owing to the international and large-scale character of the study.

### Conclusions

This large-scale international RCT examines the effectiveness of the Untire app aimed at improving fatigue and QoL in patients and survivors of cancer. To provide more insight into the process of change, the study also tests the influence of several potential mediators such as physical activity, catastrophizing, and mindfulness. Moderator analyses will further our understanding of who benefits more depending on, for example, age, gender, and disease status. These findings will further improve the theoretical notions of fatigue in relation to other variables involved in facilitating or moderating change. In terms of practical implications, the findings will enable us to further improve the Untire app and similar interventions, for example, by giving more or less weight to specific therapeutic elements.

## Appendix

Multimedia Appendix 1 Ethical review and approval by country.

Multimedia Appendix 2 Subject information for participation in scientific research.

Multimedia Appendix 3 Informed Consent.

## Abbreviations

CBT: cognitive behavioral therapy
CRF: cancer-related fatigue
eHealth: electronic health
FSI: Fatigue Symptom Inventory
GLMM: General Linear Mixed Models
HDI: Helen Dowling Institute
ITT: intention-to-treat
MBSR: Mindfulness-Based Stress Reduction
mHealth: mobile health
PROMIS-29: Patient-Reported Outcomes Measurement Information System
QoL: quality of life
RCT: randomized controlled trial
UMCG: University Medical Center Groningen
