# Supplementary material for: Beating Cancer-Related Fatigue With the Untire Mobile App: Protocol for a Waiting List Randomized Controlled Trial
Source: JMIR Res Protoc. 2020 Feb 14;9(2):e15969. doi: 10.2196/15969 (PMC7055831; doi:10.2196/15969)
Supplement: Multimedia Appendix 1 [file resprot_v9i2e15969_app1.docx]

**Multimedia Appendix 1:** Ethical review and approval by country

|  | |
| --- | --- |
| Country | Comment |
| Australia | The Research Ethics and Compliance department of Monash University stated that the ethical approval of the UMCG is sufficient, and no further ethical procedures are needed. |
| Canada | The study is not funded nor hosted in Canada. Therefore Canadian national authorities do not need to review this study ethically. |
| Netherlands | The Medical Ethical Committee of the University Medical Center Groningen gave a favorable ethical opinion for this study (METc2017/514). |
| United Kingdom | The Wales Research Ethics Committee Bangor gave a favorable ethical opinion for this study (REC: 18/WA/0003; IRAS ID: 239359). |
| United States | The Office for Human Research Protections of the Department of Human services exempted this study from ethical approval. |
