# Supplementary material for: Beating Cancer-Related Fatigue With the Untire Mobile App: Protocol for a Waiting List Randomized Controlled Trial
Source: JMIR Res Protoc. 2020 Feb 14;9(2):e15969. doi: 10.2196/15969 (PMC7055831; doi:10.2196/15969)
Supplement: Multimedia Appendix 2 [file resprot_v9i2e15969_app2.docx]

**Multimedia Appendix 2:** Subject information for participation in scientific research

| Dear Sir/Madam,  Thank you for your interest in this study on cancer-related fatigue. You are being asked to take part in a scientific study on the Untire App because you mentioned having suffered from this kind of fatigue.  Participation in the study is entirely voluntary, but because of scientific guidelines, we need to ask you for your digital consent before taking part. To help you decide whether you want to take part in the study or not, we will first explain to you how the study is conducted and what is expected from you. We kindly ask you to read this information carefully. If you decide to participate in the study after reading the information, you can give us digital consent. This can be done online. Of course, you may also decide you do not want to be part of the research. Please feel free to decide either way.  1. General information  The Untire App study is being carried out by the Department of Health Psychology of the University Medical Center Groningen, the Netherlands. This study is funded by a European Grant (Horizon 2020) appointed to Tired of Cancer BV (ToC BV). ToC BV is the organization that has developed the App, and that has requested the research team from Groningen to carry out an independent and objective study as regards the App.  In order to make this study purposeful, 6,000 participants from different countries are required. The Medical Ethical Committee of the University Medical Center Groningen has approved this study.  2. Purpose of this study  The purpose of this study is to examine whether using the training in the Untire application (hereinafter referred to as ‘the App’) can reduce cancer-related fatigue and improve quality of life. Also, the extent to which the training in the App is used will be studied. The App is based on scientifically proven methods for treating cancer-related fatigue in clinical practice. At the moment, the App is not yet available in your language. The App will first become available for smartphones.  To investigate whether the Untire App can indeed reduce cancer-related fatigue and improve quality of life, we need to compare cancer-related fatigue and quality of life experienced by subjects who use the App versus subjects who do not use the App.  3. Background of the study  Many patients and survivors of cancer suffer from severe fatigue every day. This can have a huge impact on their daily routines and quality of life, as you may well have experienced yourself.  Psychosocial treatments can reduce cancer-related fatigue, but not all doctors and patients are aware of this. Tired of Cancer BV has transformed elements of these treatments and of scientific research into training in the form of an application. The advantage of offering training via an App is that more people can have access to this form of support. They can do this independently, at their own pace, whenever and wherever they want. Although the App is based on proven methods in clinical practice, we need to investigate if the App indeed has beneficial effects for its users. Therefore, we would appreciate your participation: this study investigates whether the App can improve cancer-related fatigue and quality of life.  4. How is this study set up?  If you are willing to participate in this study, you will start by filling in a questionnaire. This takes about 20 minutes. The questions are about fatigue, factors related to fatigue, and your illness. After that, you will also receive a questionnaire after 4, 8, and 12 weeks. These questionnaires (except for the one at 12 weeks) are shorter than the first questionnaire. You are not supposed to use the App during these 12 weeks. If you complete these questionnaires after 12 weeks, and the App is available in your language, you will receive an email with an access code that gives you six months’ access to the App. Twelve weeks after receiving this code, we will send you the final, short questionnaire.  5. What is expected of you?  In order to carry out the study properly, it is important that you fill in the questionnaires that you will receive by email. Please fill in the questionnaire within one week after you have received the invitation.  6. Possible benefits and drawbacks of taking part in this study  We invite you to properly weigh up the possible benefits and drawbacks of taking part in this study before you decide to join.  Benefits may be:  • Six months’ access to the App.  • Using the App may be beneficial to your cancer-related fatigue and increase your quality of life.  Drawbacks may be:  • Filling in the questionnaires takes some time and perhaps energy.  7. If you do not want to participate or you want to stop participating in the study  You decide whether or not to participate in the study. As mentioned before, participation is entirely voluntary. If you do participate in the study, you may always change your mind and decide to stop at any time during the study. You do not have to give a reason as to why you want to stop. If you do not want to receive any further emails with questionnaires, you can send an email to the research team ***(research team email)***. Please also mention the email address that you have provided so the research team can stop sending you questionnaires. Any (anonymized) data collected until that time will still be used for the study.  8. End of the study  Your participation in the study stops when:  • you have completed all the questionnaires  • you decide to stop  • Tired of Cancer BV., the government or the Medical Research Ethics Committee decides to stop the study.  After processing the data, the results will be presented by The University of Groningen and Tired of Cancer BV.  9. Usage and storage of your data  For this study, it is necessary to collect and use your data. We will collect your data from questionnaires that you filled in, collect data you entered in the App, and collect automatically stored data regarding the use of the App. The last two are called log data.  You will receive an access code, and all log data we gather from the App is stored using this code. Personal data from the App that could identify you directly will not be linked to these data. How the App handles your data, you can read in the App’s privacy policy.  All your data will remain confidential. Members of the researcher team of the University Medical Center Groningen, the Netherlands, are the only people who will know which code you have. All data will be analyzed at group level and anonymized. The usage and storage of data meet all legal guidelines concerning privacy and data.  Apart from our research team, the monitors and auditors who check whether the study is being conducted in a proper and reliable manner will have access to the data. Your data will always be safe and will not be shared with any third parties. According to legal obligations, the researchers will store your anonymized data for 15 years.  Future use of data  We may be able to use your data for additional research in the future. This will concern research about cancer-related fatigue. You can indicate if you agree with this on the consent form. You can always withdraw this consent.  10. Study subject insurance  There are no risks for you if you take part in this study. The reviewing committee has, therefore decided that the University Medical Center Groningen does not need to take out additional insurance.  11. Compensation for participation  The Untire App is free of charge for you for six months.  12. Any questions?  If you have any questions, please take a look at the Q&A (questions and answers) first. You can also contact one of the researchers of this project (untire@umcg.nl) from the Health Psychology department of the University Medical Center Groningen, the Netherlands. If you would like any independent advice about participation in this study, you may contact ***contact name (contact email)***. She is aware of the study but is not involved in it. If you have any complaints, you may contact the complaint officer of the University Medical Center Groningen ***(officer email)***.  13. Signing the informed consent  On the next page, you can indicate that you have understood the information presented above and consent to participation in the study.  After digitally signing the informed consent, you will receive an email with a copy of this information letter and your digital consent.  Thank you very much for your time and interest in our study. |
| --- |
