# Supplementary material for: Beating Cancer-Related Fatigue With the Untire Mobile App: Protocol for a Waiting List Randomized Controlled Trial
Source: JMIR Res Protoc. 2020 Feb 14;9(2):e15969. doi: 10.2196/15969 (PMC7055831; doi:10.2196/15969)
Supplement: Multimedia Appendix 3 [file resprot_v9i2e15969_app3.docx]

**Multimedia Appendix 3:** Informed Consent

| By signing this consent form, you agree to the following statements:  •I have read the subject information form.  •I am aware that participation is voluntary. I understand that I can decide at any time not to participate after all or to withdraw from the study. I do not need to give a reason for this.  •I am aware that some people can access my data. These people are listed in this information sheet.  •I consent to my data being stored at the research location for 15 years after this study.  •I wish to participate in this study.  •☐ I agree with the statements above.  I consent to my data being used in future studies investigating cancer-related fatigue:  ☐ Yes ☐ No  I consent to be contacted again after this study for a follow-up study:  ☐ Yes ☐ No  I hereby declare that I have been fully informed about this study.  Date:__________  Name: __________  If information comes to light during the study that could affect the study participant's consent, I will inform him/her of this in a timely fashion.  Date: __________  Dr. Anne Looijmans; study coordinator  [Signature]  University Medical Center Groningen, Department of Health Psychology, the Netherlands |
| --- |
